# Synthesis of cembratriene-ol and cembratriene-diol in yeast via the MVA pathway

**DOI:** 10.1186/s12934-021-01523-4

**Published:** 2021-02-02

**Authors:** Yu Zhang, Shiquan Bian, Xiaofeng Liu, Ning Fang, Chunkai Wang, Yanhua Liu, Yongmei Du, Michael P. Timko, Zhongfeng Zhang, Hongbo Zhang

**Affiliations:** 1 TRI of CAAS-UVA Joint Laboratory of Synthetic Biology, Tobacco Research Institute, Chinese Academy of Agricultural Sciences, Qingdao, 266101 China; 2 Department of Biology, University of Virginia, 485 McCormick Road, Charlottesville, VA 22904 USA

**Keywords:** Cembranoids, Tobacco, Biosynthesis, Yeast, CBTS1, CYP450, MVA pathway

## Abstract

**Background:**

Cembranoids are one kind of diterpenoids with multiple biological activities. The tobacco cembratriene-ol (CBT-ol) and cembratriene-diol (CBT-diol) have high anti-insect and anti-fungal activities, which is attracting great attentions for their potential usage in sustainable agriculture. Cembranoids were supposed to be formed through the 2-C-methyl-d-erythritol-4-phosphate (MEP) pathway, yet the involvement of mevalonate (MVA) pathway in their synthesis remains unclear. Exploring the roles of MVA pathway in cembranoid synthesis could contribute not only to the technical approach but also to the molecular mechanism for cembranoid biosynthesis.

**Results:**

We constructed vectors to express cembratriene-ol synthase (CBTS1) and its fusion protein (AD-CBTS1) containing an N-terminal GAL4 AD domain as a translation leader in yeast. Eventually, the modified enzyme AD-CBTS1 was successfully expressed, which further resulted in the production of CBT-ol in the yeast strain BY-T20 with enhanced MVA pathway for geranylgeranyl diphosphate (GGPP) production but not in other yeast strains with low GGPP supply. Subsequently, CBT-diol was also synthesized by co-expression of the modified enzyme AD-CBTS1 and BD-CYP450 in the yeast strain BY-T20.

**Conclusions:**

We demonstrated that yeast is insensitive to the tobacco anti-fungal compound CBT-ol or CBT-diol and could be applied to their biosynthesis. This study further established a feasibility for cembranoid production via the MVA pathway and provided an alternative bio-approach for cembranoid biosynthesis in microbes.

## Background

Cembranoids are a group of natural carbocyclic diterpenes structurally composed of a 14-carbon cembrane ring. This kind of compounds were firstly identified in conifer plants and have been found to widely present in nature [1, 2]. So far, hundreds of cembranoids have been isolated from plants, insects, alligators, and marine organisms [3, 4]. Cembranoids possess multiple bioactivities, such as anti-fungal [5–7], anti-insect [8], anti-cancer [9, 10], anti-inflammatory [11–13], neuroprotection [14, 15], etc. and have great attractions not only to pharmacology but also to agrochemistry. Tobacco is the land plant most abundant in cembranoids whose abundance could significantly affect the aromatic property of tobacco [16]. Cembratriene-ol (CBT-ol) and cembratriene-diol (CBT-diol) are two major cembranoids in tobacco. And, they are synthesized and secreted by the glandular trichomes of tobacco [3].

**Fig. 1:**
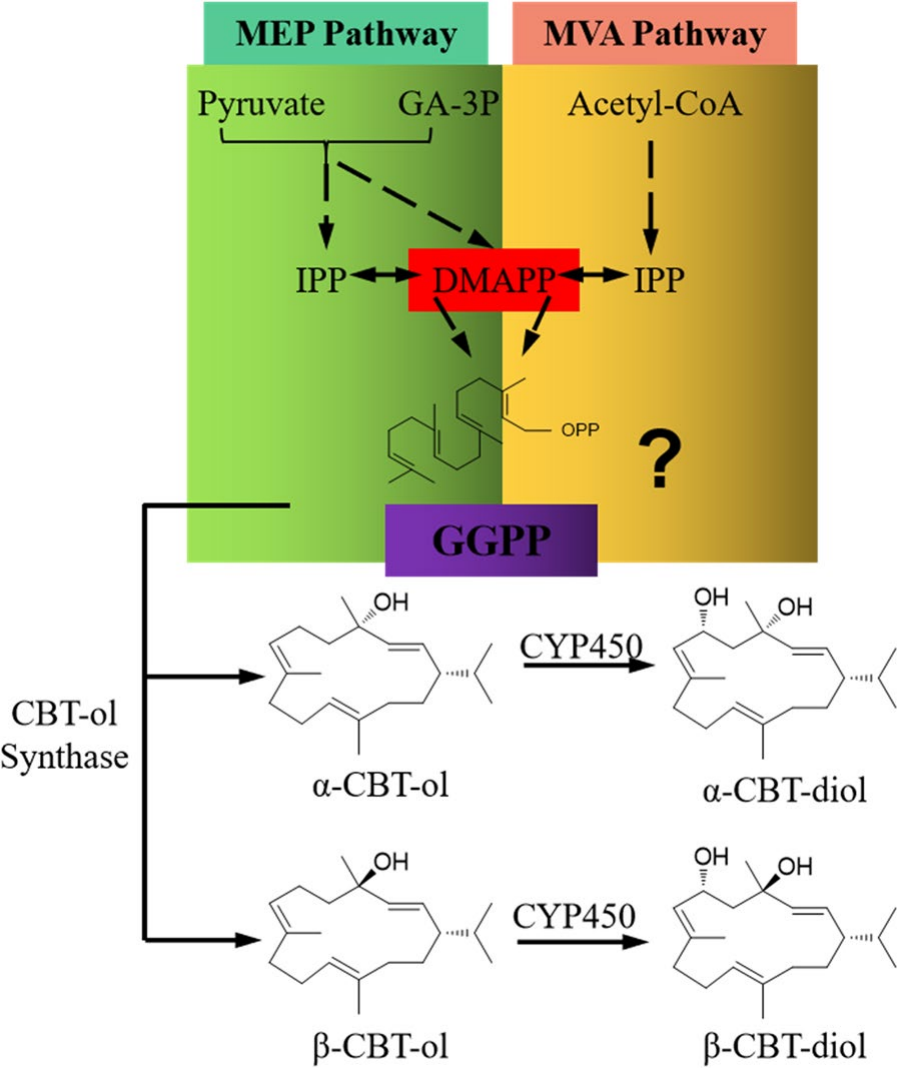
A schematic diagram of CBT-ol/CBT-diol synthesis in tobacco. Tobacco GGPP could be formed via MEP or MVA pathway. The MEP pathway derived GGPP was shown to be the precursor of CBT-ol/CBT-diol, but it is unknown whether GGPP from the MVA pathway could act as their precursor. Formation of CBT-ol from GGPP is catalyzed by CBTS1, and further synthesis of CBT-diol is catalyzed by CYP450. Both CBT-ol and CBT-diol have two structural isomers

As one type of diterpenoids, tobacco CBT-ol and CBT-diol are derived from geranylgeranyl diphosphate (GGPP) under the sequential catalyzation by CBTS1 (cembratriene-ol synthase) and CYP450 (cytochrome P450 hydroxylase) (Fig. 1) [17–19]. And, CBT-ol and CBT-diol each have two structural isomers (i.e., α and β isomers) [18–20]. In plant, GGPP are synthesized through two common biological pathways, i.e., the mevalonate (MVA) pathway that occurs in the cytoplasm of eukaryotes [8, 18, 21, 22] and the 2-C-methyl-d-erythritol-4-phosphate (MEP) pathway that presents in the plastids [23]. In the MVA pathway, MVA is derived from acetoacetyl-CoA, which is formed by condensation of two molecules of acetyl-coenzyme A (Acetyl-CoA), under the catalyzation by 3-hydroxy-3-methylglutaryl synthase (HMGS) and HMG-CoA reductase [24]. Then, MVA is converted through MVA 5-diphosphate to isopentenyl diphosphate (IPP), and the IPP is converted to dimethylallyl pyrophosphate (DMAPP). IPP and DMAPP are catalyzed by geranylgeranyl pyrophosphate synthetase (GGPPS) to produce GGPP for terpenoid synthesis [24]. In the start of MEP pathway, the pyruvate-derived (hydroxyethyl) thiamin and the C1 aldehyde group of d-glyceraldehyde 3-phosphate (GA-3P) are condensed to generate 1-deoxy-d-xylulose-5-phosphate (DXP), which is then converted to methylerythritol 4-phosphate (MEP), then MEP is catalyzed in sequential steps to form 4-hydroxy-3-methylbut-2-enyldiphosphate (HMBPP). Eventually, HMBPP is reduced to IPP and DMAPP [25, 26], which act as general precursors for terpenoid formation and can be catalyzed to produce the general precursors GGPP for diterpenoids, such as CBT-ol [27].

Previous studies demonstrated that tobacco CBT-ol and CBT-diol have high anti-insect and anti-fungal activities [5–7], respectively, and their potential application in sustainable agriculture is expected in near future. Even though their application in agricultural is greatly anticipated [8, 28, 29], it is currently limited by the high cost of preparation from natural resources. The production of CBT-ol and CBT-diol using tobacco plants is of high cost and is far from meeting the commercial demands [30, 31]. The chemical approaches for cembranoid synthesis have not been established so far, thus their industrial production is unable to be realized in a short period. Furthermore, chemical synthesis always yields high environmental pollution, which goes against the concept of sustainable agriculture. On the other hand, metabolic engineering of microbes for synthesizing natural plant products has recently made a great progress [32–36], which provides a way to produce natural compounds via fermentation method [27]. And, a number of terpenoid compounds have been successfully synthesized in metabolic engineered bacteria or fungi, such as artemisinic acid [37], tanshinones [38, 39], resveratrol [40], ginsenoside [41]. Therefore, the biosynthetic approach is a practicable way to produce cembranoids under mild condition with lower cost.

In previous studies, tobacco cembranoids were hypothesized to be formed through the MEP pathway, and the bioengineered synthesis of CBT-ol was achieved in *Escherichia coli* via the metabolic engineered MEP pathway [29]. Whether there is a possibility for synthesizing tobacco cembranoid via the MVA pathway remains unknown. Exploring the roles of MVA pathway in cembranoid synthesis could contribute not only to the technical approach but also to the molecular mechanism for cembranoid biosynthesis.

## Materials and methods

### Determination of the effect of tobacco cembranoids on *Saccharomyces cerevisiae* and *Botrytis cinerea* growth

To determine the growth effects of tobacco cembranoids on *Saccharomyces cerevisiae*, YPD agar medium (1% yeast extract, 2% peptone, 2% glucose, 1.5% agar) plates with the indicated amount of CBT-ol or CBT-diol were prepared, and a cell suspension of *S. cerevisiae* BY4742 (OD600 = 0.2) was spread onto the plates by 0.5 mL/plate. Plates free of CBT-ol or CBT-diol were prepared as mock treatment control by adding the same volume of pure solvent (i.e., 95% ethanol) as that for CBT-ol and CBT-diol plates, and inoculated with the equal amount of yeast cells. After 4 days of cultivation at 30 ℃ incubator, the growth of yeast cells was observed and photographed.

To determine the effect of tobacco cembranoids on *B. cinerea*, PDA (Potato Dextrose Agar) medium plates with indicated amount of CBT-ol or CBT-diol were prepared, and a cube (⌀ = 5 mm) of *B. cinerea* mycelium was inoculated onto each plate. Plates for mock treatment control were prepared in a similar method as described above and inoculated with mycelium cube. After 4–6 days of cultivation at 26 ℃ incubator, the growth of *B. cinerea* was observed and photographed. CBT-ol and CBT-diol for preparing the YPD agar and PDA plates were isolated from tobacco trichomes as previously described [28].

### Vector construction and yeast transformation

In order to express the tobacco cembranoid synthase CBTS1 (GenBank: AAS46038.1) in yeast, the corresponding gene sequence was codon-optimized (Additional file 1: Table S1) and fully synthesized by Sangon Biotech (China). To construct pGADT7-CBTS1-His vector for expressing CBTS1 (GenBank: AAS46038.1), the corresponding DNA fragment was amplified using CloneAmp™ HiFi PCR Premix (Takara, Japan) with specific primers and then cloned by In-Fusion^®^ (Takara, Japan) cloning method into a vector modified from pGADT7 (Clontech, USA) by deleting the AD (GAL4 activation domain) region, and a fragment encoding 6× His tag was introduced to the downstream of *CBTS1* for protein detection by Western Blot via the primers for gene amplification. And, the gene fusion encoding CBTS1-His was set under the control of ADH1 promoter in the modified vector. To construct pGADT7-CBTS1 vector, the synthesized *CBTS1* gene was cloned into the original pGADT7 vector (Clontech, USA) to express a fusion protein of AD-CBTS1, in which AD serves as an expression leader for enhancing the protein expression level in yeast. In a similar method, the gene sequence of *CYP450* (GenBank: AF166332) was synthesized after codon-optimization (Additional file 1: Table S1) and cloned into pGBKT7 vector to express a fusion protein of BD-CYP450, in which BD (GAL4 DNA binding domain) serves as an expression leader for expression of CYP450 in yeast. The primers used for gene amplification and vector construction are listed in Additional file 1: Table S2.

The derived vectors were introduced into the indicated yeast strains, including BY4742 (*MATα, his3Δ1, leu2Δ0, lys2Δ0, MET15, ura3Δ0*) [42], BY-T1 (*MATa, trp1Δ, his3Δ1, leu2Δ0, lys2Δ0, MET15, ura3Δ0, δDNA::P*_*PGK1*_*-tHMG1-T*_*ADH1*_*-P*_*TEF1*_*-LYS2-T*_*CYC1*_) [43], and BY-T20 (*MATα, trp1Δ0, leu2Δ0, ura3Δ0, trp1::HIS3-P*_*PGK1*_*-BTS1/ERG20-T*_*ADH1*_*-P*_*TDH3*_*-SaGGPS-T*_*TPI1*_*-P*_*TEF1*_*-tHMG1-T*_*CYC1*_) [44] for required protein expression assays as well as CBT-ol and CBT-diol synthesis. And, the yeast transformants were selected by cultivation on SD (synthetic defined) medium plates with desired dropout (DO) supplements (Takara, Japan).

### Detection of yeast expressed CBTS1 protein by Western Blot

Western Blot assay was applied to determine the expression of 6× His-tagged and AD-tagged fusion proteins of CBTS1 in yeast cells using SDS-PAGE gel for protein isolation. The CBTS1-His protein was detected with an HRP-conjugated mouse anti His-tag antibody (CoWin Biosciences, China) and visualized by ECL (enhanced chemiluminescence) method, with a protein Marker containing 6× His-tagged proteins (Sangon Biotech, C510010-0500) as positive control. The AD-CBTS1 protein was detected with a mouse anti AD-tag primary antibody (Clontech, USA) and an HRP-labeled goat anti-mouse IgG secondary antibody for ECL visualization, with yeast expressing AD only as control.

### Yeast cultivation and cembranoid extraction

The positive colonies of yeast transformant were inoculated into 5 mL liquid SD/-Leu medium (for yeast expressing CBTS1) or SD/-Leu/-Trp medium (for yeast expressing both CBTS1 and CYP450), and cultured at 30 ℃, 220 r/min for 48 h as the seed culture. For shake-flask cultivation, the seed culture was inoculated into YPD liquid medium (2% glucose, 1% yeast extract, 2% peptone) or the optimized medium (15% glucose, 1.5% yeast extract, 3% peptone, 1% KH_2_PO4) at a ratio of 5%, and cultured at 30 °C and 220 r/min for the indicated time period [45]. For bioreactor cultivation, the seed culture was inoculated into a bioreactor containing 1 L of optimized medium mentioned above and cultured at 30 °C, with the dissolved oxygen above 30% of atmospheric oxygen and the pH maintained at 5.8. Concentrated glucose solution (80%, w/v) was fed periodically to provide adequate carbon source.

For yeast growth monitoring and cembranoid extraction, 1 L of each cell culture was collected at the indicated cultivation period and centrifuged to separate yeast cells and the cultivation broth. The yeast cells were weighed and then grounded into fine powder in liquid nitrogen, dissolved in 20 mL ddH_2_O, and further lysed for 20 min in an ultrasonic cell disruptor. The cell lysate was extracted three times with the equal volume of ethyl acetate for 30 min at 30 ℃ with agitation. After centrifugation, the upper organic phase of each extraction was collected and combined for further concentration. The cultivation broth was directly extracted with ethyl acetate in the same method. The extract was dried in a rotary evaporator at 40 ℃, and dissolved in 5 mL of ethyl acetate for further analyses.

### Determination of CBT-ol and CBT-diol by GC–MS

The cembranoid extract obtained by above method was dried in nitrogen flow, and then dissolved in 1 mL ethyl acetate for GC–MS assay. In GC–MS assay, the sample was loaded onto the HP-5 ms column (30 m × 250 μm × 0.25 μm) of a HP7890B Gas Chromatography coupled with HP5977A Mass Spectrometer (Agilent, USA). The column temperature was initially set at 80 ℃ and maintained for 1 min, and increased to 200 ℃ at a temperature gradient of 15 ℃/min to maintain for another 1 min. Then, the column temperature was increased to 240 ℃ by 4 ℃/min and kept for additional 2 min. The mass spectra were acquired in the m/z 50–650 range at 70 eV (EI) using negative ionization mode. CBT-ol and CBT-diol were identified by comparing their retention times and mass spectra with those of the standards and the mass spectra data at NIST Database.

### Measurement of CBT-ol and CBT-diol by UPLC

For UPLC assay, the cembranoid extract was dried in nitrogen flow and then dissolved in 1 mL of 70% acetonitrile. In the UPLC assay, 5 µL sample was injected into ultra-performance liquid chromatography (UPLC; Waters, USA) under following optimized conditions: BEH C18 column (1.7 µm, 2.1 mm × 100 mm) with the column temperature of 40 °C, a gradient mobile phase as indicated in Additional file 1: Table S3 at the flow rate of 0.3 mL/min, and a UV detector for detection of CBT-ol and CBT-diol at 208 nm. The lab-available authentic standards CBT-ol and CBT-diol, which were isolated and purified from a 95% EtOH extract of tobacco trichomes using a preparative HPLC system (Waters Technologies Ltd., USA) equipped with a ultraviolet–visible light detector and a Prep C18 OBD (19 mm × 250 mm column, 10 μm) as previously described [28], were used to distinguish the corresponding peaks in the UPLC chromatograms of the samples.

## Results and discussion

### Feasibility of *S. cerevisiae* as the host strain for cembranoid synthesis

**Fig. 2:**
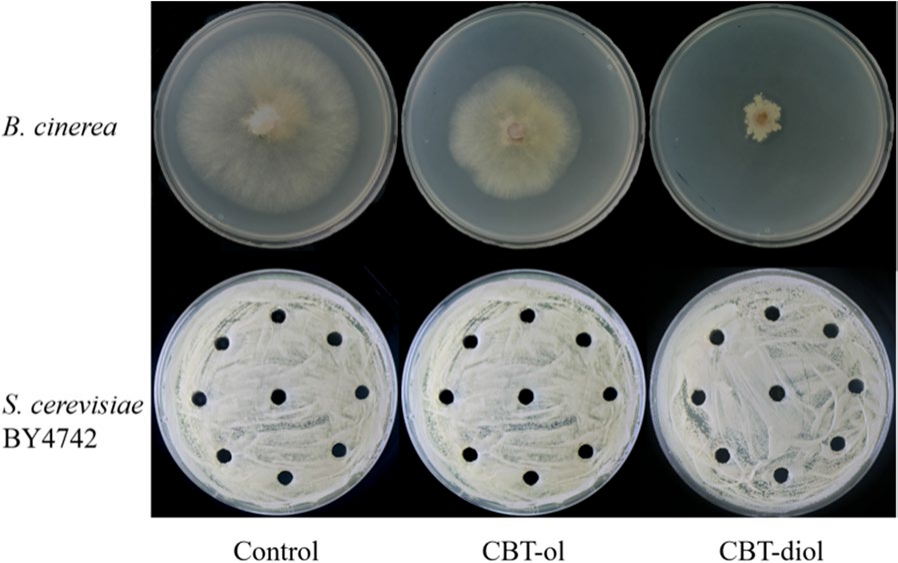
Growth of *B. cinerea* and *S. cerevisiae* on the medium plates with mock treatment CBT-ol, or CBT-diol

As mentioned above, tobacco cembranoids possess highly active anti-fungal activities against mold or mildew fungus [26, 28], and may limit the construction of fungal system for cembranoid synthesis. Whereas, their effects on yeast (a type of fungus) are still unknown. To explore the possibility of synthesizing tobacco cembranoids in yeast, we examined the growth inhibitory effects of tobacco CBT-ol and CBT-diol on *S. cerevisiae* BY4742 [42] with mold fungus *B. cinerea* as control. The results showed that the growth of *B. cinerea* on PDA (Potato Dextrose Agar) plate was extremely suppressed by tobacco CBT-diol at 200 μM comparing to control on the plate without CBT-diol, and tobacco CBT-ol displayed a much weaker suppression on the growth of *B. cinerea* than CBT-diol (Fig. 2). However, neither CBT-diol nor CBT-ol exhibited an observable suppressive effect on the growth of yeast (Fig. 2). These findings suggest that tobacco CBT-diol may function with different patterns in yeast compared to their action in the mold fungus *B. cinerea*, and that yeast could be adopted as a fungal host for synthesizing tobacco CBT-ol and CBT-diol.

### Expression of cembranoid synthase CBTS1 in yeast

**Fig. 3:**
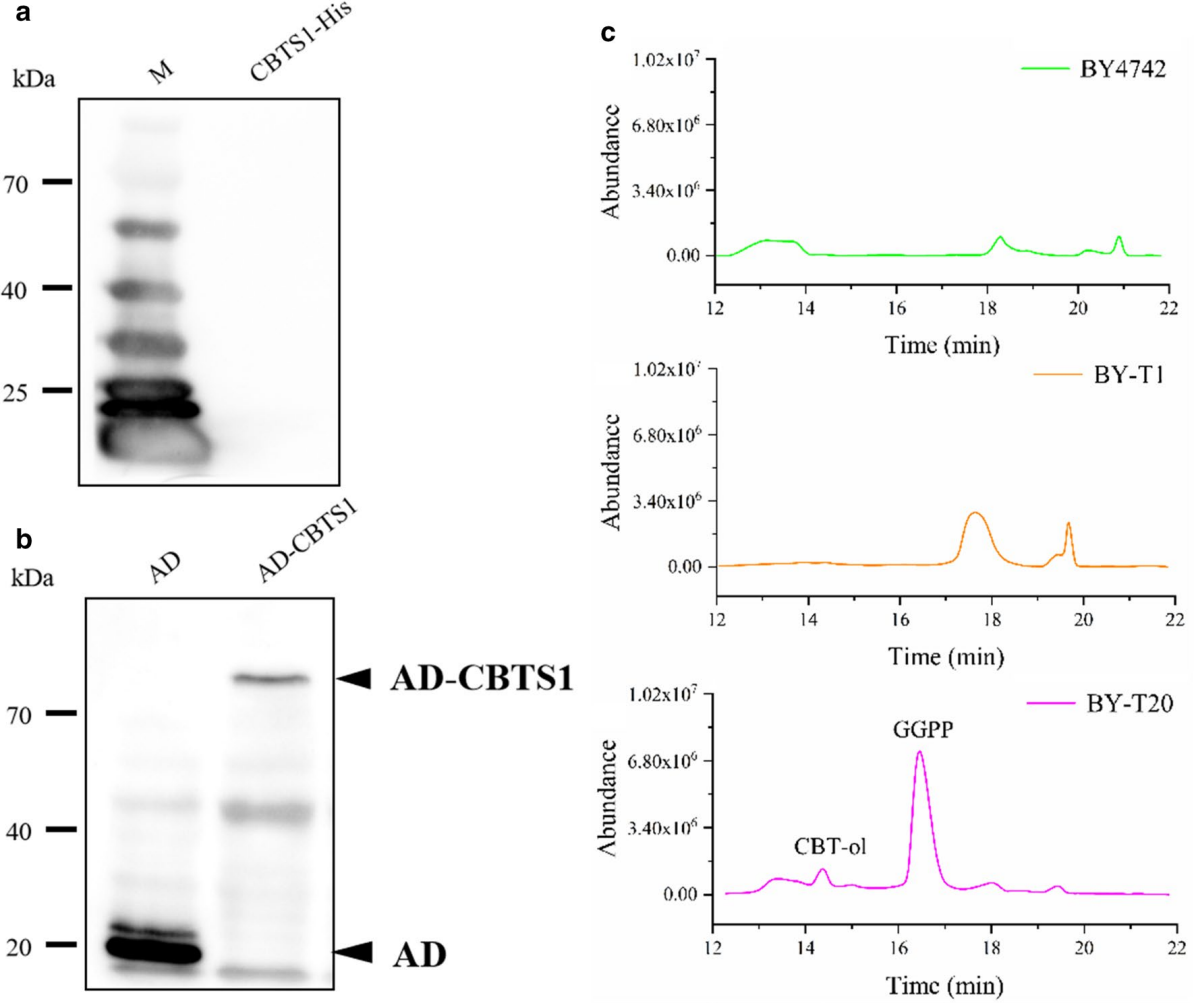
Production of CBT-ol in yeast. **a** Detection of CBTS1-His protein by Western Blot with anti-6× His antibody. M indicates the lane of protein Marker which contains 6× His-tagged proteins and serves as a positive control. Labels at left indicate the molecular weights. **b** Detection of AD-CBTS1 protein by Western Blot with anti-AD antibody. Labels at left indicate molecular weights, and arrows at right show the proteins of AD-CBTS1 and AD only. **c** GC–MS spectrums of extracts from AD-CBTS1-expressiong yeast strains BY4742, BY-T1 and BY-T20, respectively. The GC–MS peaks for CBT-ol and GGPP are indicated, and those of unidentified compounds are unlabeled

To construct vector for expressing tobacco cembratriene-ol synthase CBTS1 (GenBank accession: AAS46038.1) in yeast, the corresponding gene sequence was fully synthesized after codon-optimization for yeast expression, which was firstly cloned under the control of ADH1 promoter in a vector modified from pGADT7 (Clontech, USA) by deletion of the GAL4 AD domain fragment. And, a 6× His-tag was placed at the C-terminal of CBTS1 for protein detection (Additional file 1: Figure S1). However, no protein expression could be detected by Western Blot when the obtained vector pGADT7-CBTS1-His was introduced into yeast (Fig. 3a). To conquer the protein expression problem, we then cloned *CBTS1* into the original pGADT7 vector to express a CBTS1 fusion protein with AD domain at the N-terminus to act as an expression leader (Additional file 1: Figure S1). Western Blot showed that the fusion protein AD-CBTS1 was successfully expressed in yeast strain BY-T20 with the obtained vector pGADT7-CBTS1 (Fig. 3b), suggesting that the addition of an N-terminal expression leader is helpful to improved the protein expression level of CBTS1 in yeast. Thus, this vector was employed for production of CBT-ol or CBT-diol in yeast. Using a similar method, the codon-optimized *CYP450* (GenBank accession: AF166332) gene was synthesized and cloned into pGBKT7 vector (Clontech, USA) to express BD-CYP450 fusion protein in yeast for CBT-diol production. The constructed vector was designated as pGBKT7-CYP450.

### Selection of yeast strain for cembranoid synthesis

Tobacco cembranoids are derived from the terpenoid precursor GGPP which may be produced via MEP pathway or MVA pathway in plants (Fig. 1). To develop microbes for tobacco cembranoid synthesis through the MVA pathway, the yeast strain BY4742 and its engineered strains BY-T1 and BT-T20 were utilized to develop the cembranoid synthetic systems. BY-T1 is a yeast strain expressing a truncated HMG-CoA reductase gene (tHMG1) to increase the upstream carbon flux to MVA pathway [43], and BY-T20 is a yeast strain with high efficiency GGPP production by expressing an engineered gene module composed of tHMG1, BTS1-ERG20 gene fusion and SaGGPS (GGPS from *Sulfolobus acidocaldarius*) [44]. Initially, pGADT7-CBTS1 vector was introduced into the yeast strain BY4742, BY-T1 and BY-T20 respectively for tobacco CBT-ol synthesis. And, 1 L of 50 h shake-flask culture for each yeast strain was subjected to CBT-ol extraction and chromatography assays using GC–MS (gas chromatography–mass spectroscopy). The results showed that yeast strain BY-T20 harboring the AD-CBTS1-expressing vector could produce CBT-ol (Fig. 3c; Additional file 1: Figure S2A), while the production of CBT-ol was undetectable in strain BY4742 or BY-T1 harboring the same vector (Fig. 3c). In accordance, a strong peak for GGPP was detected in the sample from BY-T20 harboring the AD-CBTS1-expressing vector by GC–MS assay, while no corresponding peak could be detected in the sample from BY4742 or BY-T1 harboring the same vector (Fig. 3c; Additional file 1: Figure S2B). These evidences suggest that tobacco CBT-ol could be synthesized through the MVA pathway in yeast BY-T20 by expressing CBTS1, and the abundance of GGPP in yeast is a key factor manipulating the formation of CBT-ol.

### Production of CBT-ol in yeast strain BY-T20

**Fig. 4:**
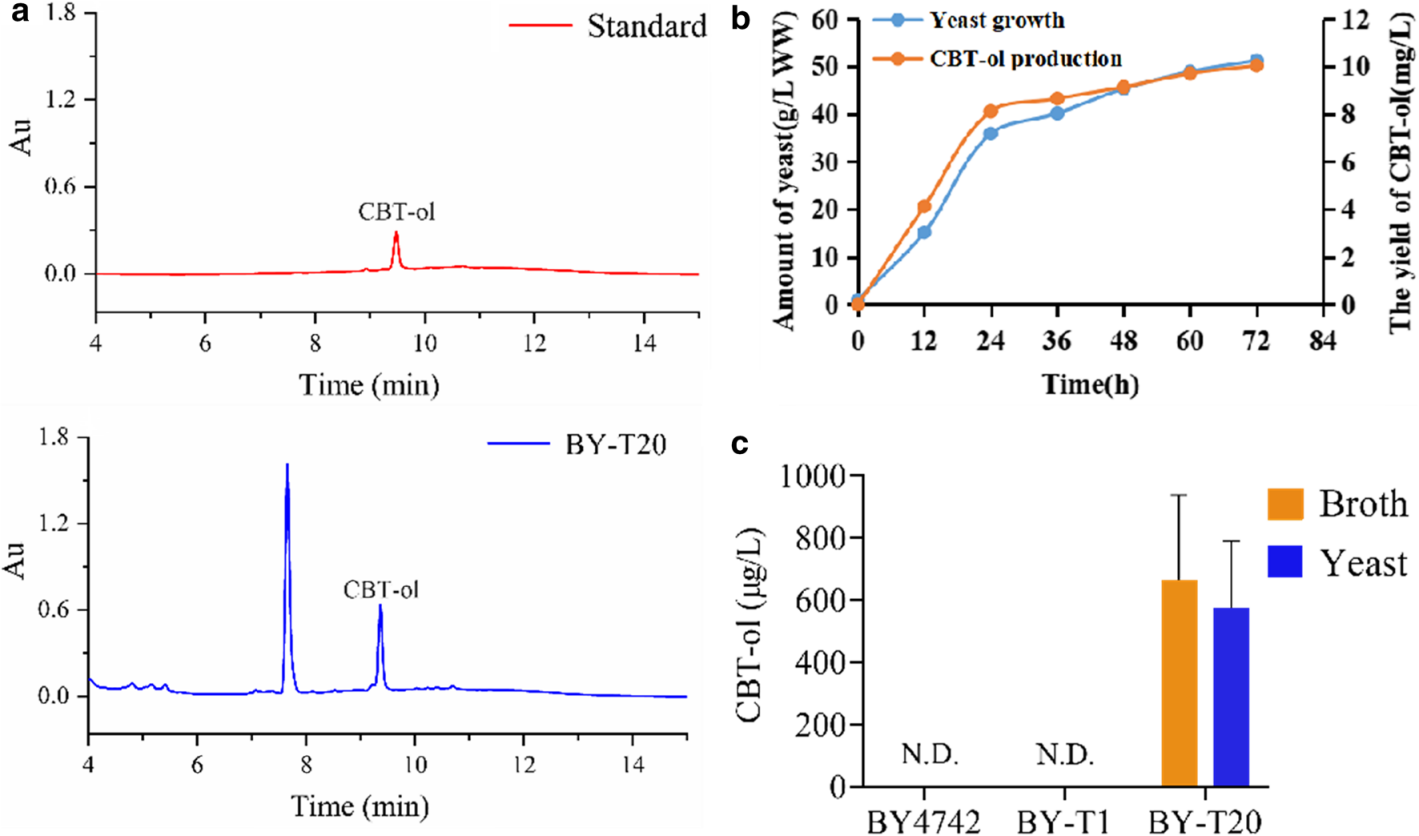
Production of CBT-ol in AD-CBTS1-expressing yeast strains BY-T20. **a** UPLC detection of CBT-ol in the extract of yeast culture. The peak for CBT-ol is indicated. **b** Yeast growth and CBT-ol production by bioreactor cultivation in the optimized medium. *WW*  wet weight. **c** Content of CBT-ol in the indicated yeas cells or cultivation broth. Each values is the average of triplicates. N.D. indicates not detected, and bars indicate mean + SD

To measure the CBT-ol production in yeast, a standard curve was plotted based on the UPLC (ultra-performance liquid chromatography) detection data from a serial dilutions of CBT-ol standard, which was isolated and purified from tobacco trichomes [28], and the CBT-ol content of the samples was determined according this standard curve. Figure 4a shows the UPLC spectra of CBT-ol standard and that for the extract from AD-CBTS1-expressing yeast. The yeast growth and CBT-ol production were monitored through a 72 h shake-flask cultivation in YPD medium or the optimized medium. And, the yeast growth could reach a maximal yield after 60 h of cultivation (Additional file 1: Figure S3A). The production of CBT-ol was determined by measuring its content in both yeast cells and the cultivation broth from 1 L yeast culture. The results turned out that the yield of CBT-ol in AD-CBTS1-expressing BY-T20 was 692.73 μg/L in cells and 863.95 μg/L in cultivation broth, accounting for a total production of about 1.56 mg/L (Fig. 4c). Interestingly, a considerable amount of GGPP still presents in the AD-CBTS1 expressing yeast BY-T20 (Fig. 3c; Additional file 1: Figure S2B), and higher CBT-ol yield is expected by further optimization of the CBTS1 enzyme. A bioreactor cultivation was also carried out to determine the yeast growth and CBT-ol production with the same yeast expression system, which produced over 50 g/L wet cells 10.02 mg/L of CBT-ol production in a 72 h of fermentation (Fig. 4b). And, the CBT-ol production is highly correlated with the yeast yield.

### Production of CBT-diol in yeast strain BY-T20

**Fig. 5:**
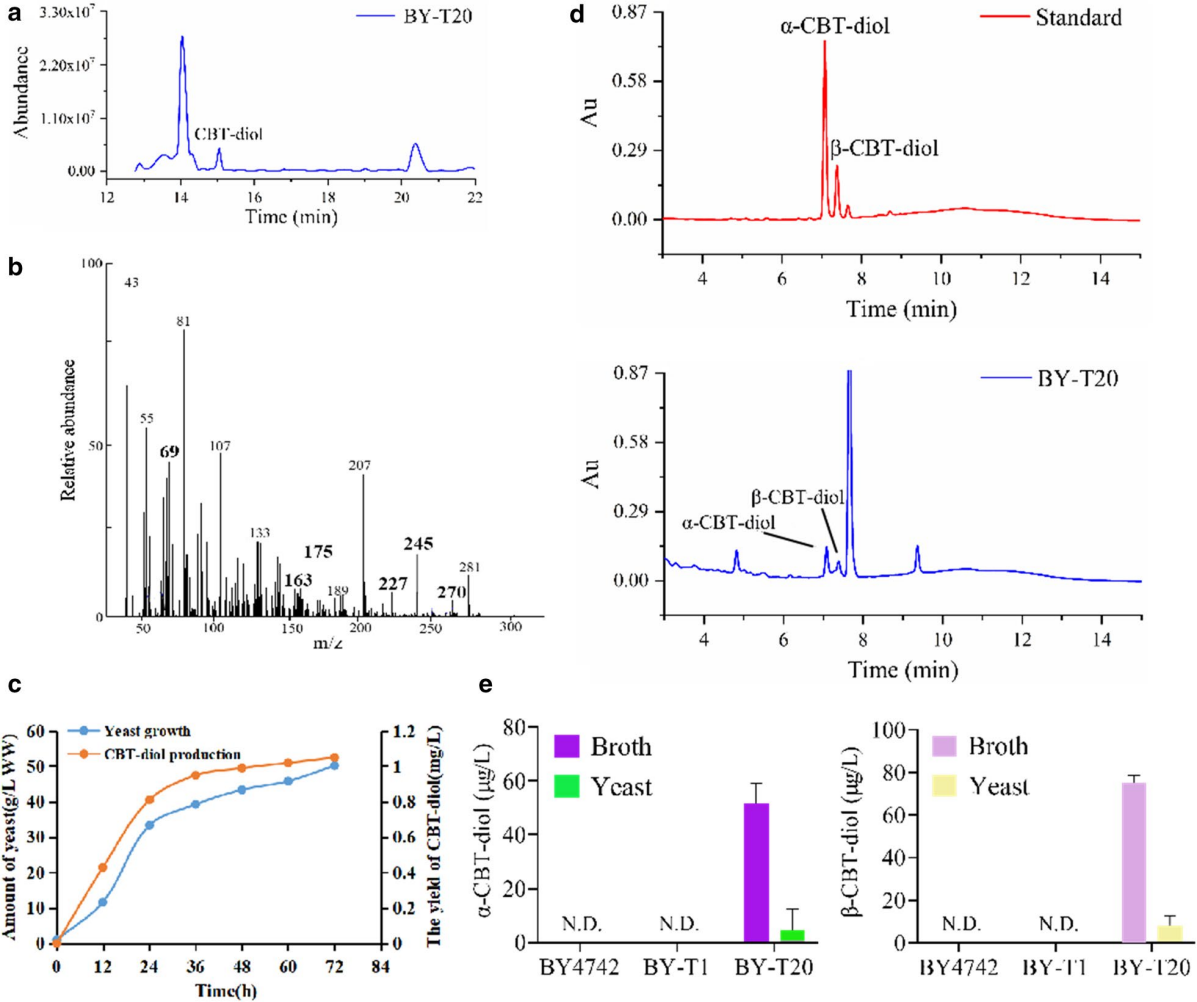
Production of CBT-diols in yeast strain BY-T20 expressing AD-CBTS1 and BD-CYP450. **a** GC–MS spectrums of yeast extract. The GC–MS peak for CBT-diol is indicated. **b** The associated mass peaks of the yeast produced CBT-diol. Bold numbers indicate the specific mass peaks discriminating CBT-diol from CBT-ol. **c** Yeast growth and CBT-diol production by bioreactor cultivation in the optimized medium. *WW* wet weight. **d** UPLC detection of CBT-diols in the extract of yeast culture. Peaks for α-CBT-diol and β-CBT-diol are indicated. **e** Content of CBT-diols in the indicated yeast culture or cultivation broth. Each values is the average of triplicates. N.D. indicates not detected, and bars indicate mean + SD

Subsequently, the production of CBT-diol in yeast was investigated by introducing both AD-CBTS1 and BD-CYP450 into the yeast strain BY-T20. And, the GC–MS analysis showed a successful synthesis of CBT-diol (Fig. 5a, b). The production of CBT-diol was also determined by measuring its content in both cells and cultivation broth of 1 L yeast culture. Monitoring the yeast growth and CBT-diol production through a 72 h shake-flask cultivation in YPD medium or the optimized medium revealed that the growth of AD-CBTS1 and BD-CYP450 co-expressing yeast BY-T20 could reach the maximal yield after 50 h of cultivation (Additional file 1: Figure S3B). In the UPLC analysis, two peaks consistent with the retention time of α-CBT-diol and β-CBT-diol standards were detected in the sample from yeast co-expressing AD-CBTS1 and BD-CYP450 (Fig. 5d), showing the production of both α-CBT-diol and β-CBT-diol. To measure the production of CBT-diol, a standard curve was constructed based on the UPLC detection data from a serial dilutions of CBT-diol, which was isolated and purified from tobacco trichomes [28], and was applied to the determination of CBT-diol content in the samples. The results showed that the production of α-CBT-diol was 13.72 μg/L in cells and 50.10 μg/L in cultivation broth, and that the production of β-CBT-diol was 13.05 μg/L in cells and 72.76 μg/L in cultivation broth. Thus, the total production of CBT-diol was about 0.15 mg/L (Fig. 5e). Further bioreactor cultivation resulted a production of over 50 g/L wet cells and 1.05 mg/L of CBT-diol in a 72 h fermentation (Fig. 5c).

## Conclusion

This study shows that tobacco CBT-ol and CBT-diol, which are anti-fungal compounds, have no observable suppressive effect on the yeast growth, and that yeast could be used for synthesizing cembranoids. And, a new method to synthesize tobacco CBT-ol and CBT-diol via the MVA pathway in yeast was further established. The biosynthesized CBT-diol showed different distribution patterns in yeast cells and the cultivation broth, which may be resulted from their different solubility or cell transportation. Currently, the production of CBT-ol and CBT-diol in yeast is 1.56 mg/L and 0.15 mg/L under shake-flask cultivation and 10.02 mg/L and 1.05 mg/L by bioreactor cultivation, respectively. But, greater yield is expected by improving enzyme expression level and strengthening the enzymatic activity. In conclusion, this work has demonstrated a feasibility for cembranoid production via the MVA pathway and established an alternative bio-approach for synthesizing tobacco cembranoids, which may promote their application in sustainable agriculture and other aspects.

## Supplementary Information

**Additional file 1: Figure S1.** Diagrams of vector structures. **Figure S2.** Mass spectra of CBT-ol and GGPP. **Figure S3.** Yeast growth and cembranoid production by shake-flask cultivation. **Table S1.** Codon-optimized sequences of *CBTS1* and *CYP450*. **Table S2.** Primers for vector construction. **Table S3.** Gradient mobile phase for UPLC assay.

## Data Availability

All material listed in the manuscript is available from the corresponding author.

## Abbreviations

CBT-ol: Cembratriene-ol
CBT-diol: Cembratriene-diol
MEP: 2-C-methyl-d-erythritol-4-phosphate
MVA: Mevalonate
GGPP: Geranylgeranyl diphosphate
CBTS1: Cembratriene-ol synthase
CYP450: Cytochrome P450 hydroxylase
Acetyl-CoA: Acetyl-coenzyme A
HMGS: 3-Hydroxy-3-methylglutaryl synthase
IPP: Isopentenyl diphosphate
DMAPP: Dimethylallyl pyrophosphate
GA-3P: d-glyceraldehyde 3-phosphate
DXP: 1-Deoxy-d-xylulose-5-phosphate
HMBPP: 4-Hydroxy-3-methylbut-2-enyldiphosphate
GC–MS: Gas chromatography–mass spectroscopy
UPLC: Ultra-performance liquid chromatography
