# Supplementary material for: Synthesis of cembratriene-ol and cembratriene-diol in yeast via the MVA pathway
Source: Microb Cell Fact. 2021 Feb 2;20:29. doi: 10.1186/s12934-021-01523-4 (PMC7852193; doi:10.1186/s12934-021-01523-4)
Supplement: Supplementary file 1 — Additional file 1: Figure S1. Diagrams of vector structures. Figure S2. Mass spectra of CBT-ol and GGPP. Figure S3. Yeast growth and cembranoid production by shake-flask cultivation. Table S1. Codon-optimized sequences of CBTS1 and CYP450. Table S2. Primers for vector construction. Table S3. Gradient mobile phase for UPLC assay. [file 12934_2021_1523_MOESM1_ESM.docx]

**Synthesis of cembratriene-ol and cembratriene-diol in yeast**

**via the MVA pathway**

Yu Zhang,^a^ Shiquan Bian,^a^ Xiaofeng Liu,^a^ Ning Fang,^a^ Chunkai Wang,^a^ Yanhua Liu,^a^ Yongmei Du,^a^ Michael P. Timko,^b^ Zhongfeng Zhang,*^,a^ and Hongbo Zhang*^,a^

^a^TRI of CAAS-UVA Joint Laboratory of Synthetic Biology, Tobacco Research Institute, Chinese Academy of Agricultural Sciences, Qingdao 266101, China;

^b^Department of Biology, University of Virginia, Charlottesville, VA 22904, USA.

^*^To whom correspondence should be addressed: E-mail: [zhanghongbo@caas.cn](mailto:zhanghongbo@caas.cn); or [zhangzhongfeng@caas.cn](mailto:zhangzhongfeng@caas.cn)

**Additional File 1:**

**
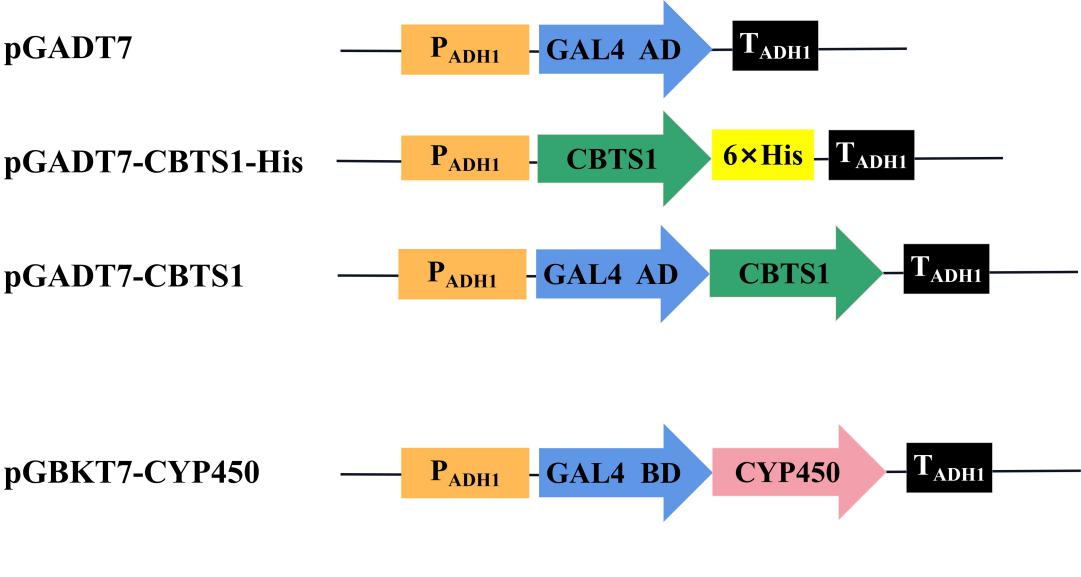
**

**Figure S1.** Diagrams of vector structures

P_ADH1_, ADH1 promoter; GAL4 AD, GAL4 activation domain region; GAL4 BD, GAL4 DNA binding domain region; T_ADH1_, ADH1 terminator.


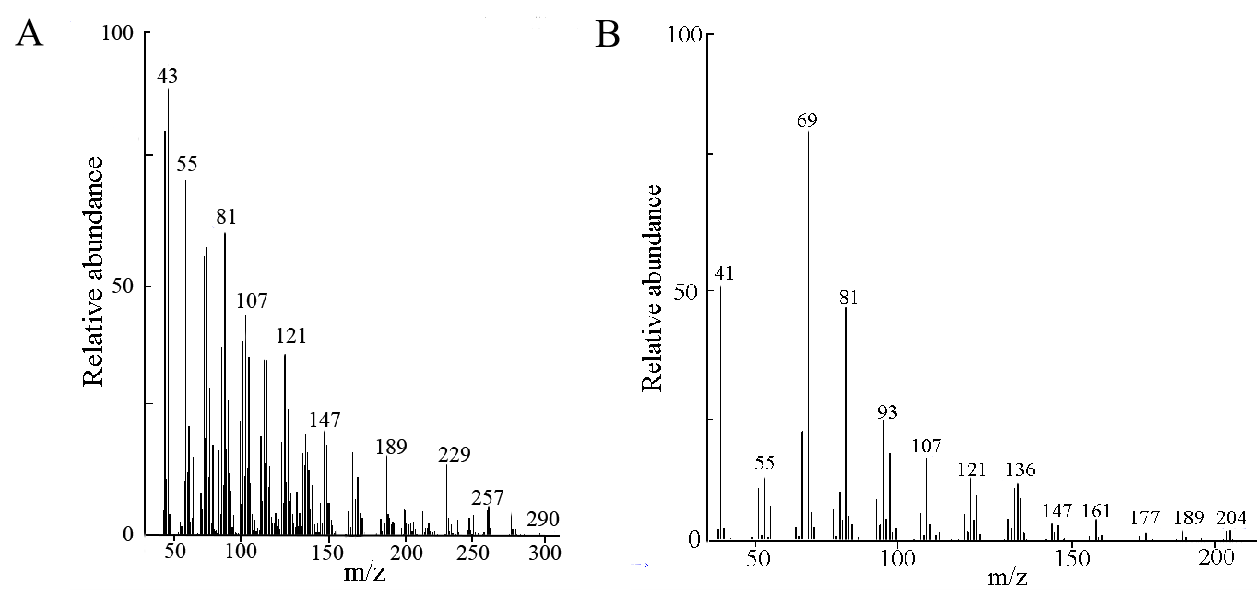


**Figure S2.** Mass spectra of CBT-ol and GGPP

1. The associated mass peaks of the yeast produced CBT-ol. (B) The associated mass peaks of the yeast produced GGPP.


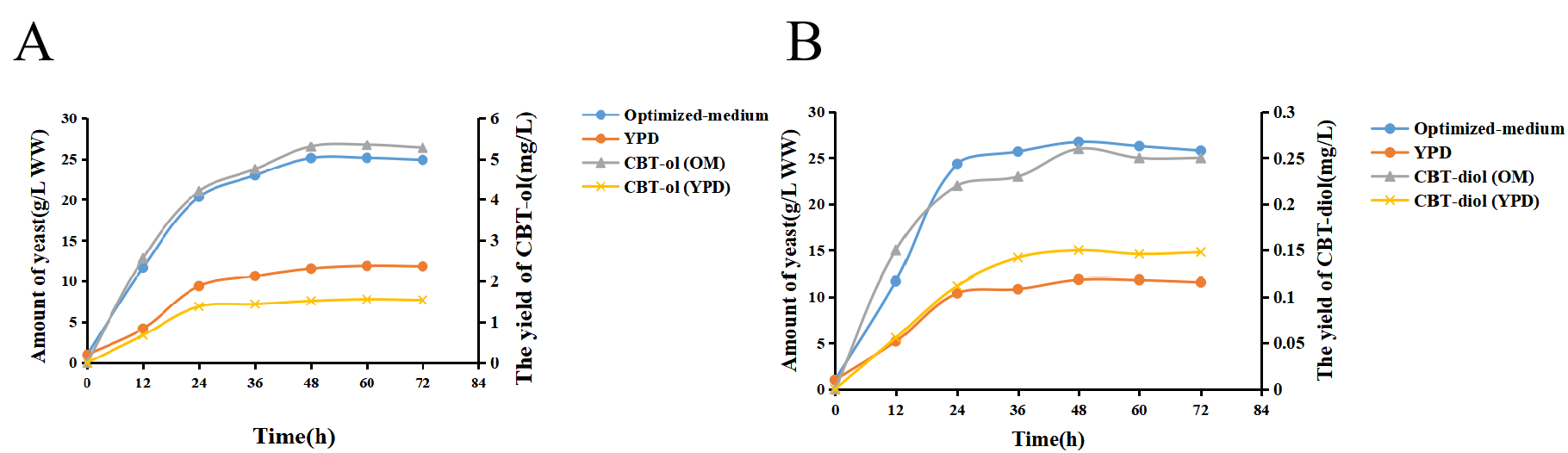


**Figure S3.** Yeast growth and cembranoid production by shake-flask cultivation

1. Yeast growth and CBT-ol production when cultured in YPD medium or the optimized medium. (B) Yeast growth and CBT-diol production when cultured in YPD medium or the optimized medium. OM in the brackets indicates optimized medium. WW=wet weight.

**Table S1.** Codon-optimized sequences of *CBTS1* and *CYP450*

| **Codon optimized sequence of *CBTS1*:**  ATGTCTCAATCTATTTCTCCTCTTATTTGTTCACATTTTGCTAAGTTTCAATCTAATATTTGGAGGTGTAATACTTCACAACTTAGAGTTATTCATTCTTCTTATGCTTCTTTTGGAGGTAGAAGAAAGGAAAGGGTTAGAAGGATGAATAGAGCTATGGATCTTTCTTCTTCTTCAAGGCACTTAGCTGATTTTCCTTCTACAATTTGGGGAGATCATTTTCTATCATATAATTCTGAAATTACTGAAATAACTACTCAAGAAAAGAATGAGCATGAAATGCTTAAGGAAATTGTTAGAAAGATGCTTGTTGAAACACCTGATAATAGTACACAAAAACTTGTTTTGATTGATACAATTCAAAGACTTGGTTTGGCTTATCATTTTAATGATGAGATTGAAAATTCAATTCAAAATATTTTTAATTTGTCACAAAATAGTGAGGATGATGATGAGCATAACCTTTATGTTGCTGCATTGAGGTTTAGACTTGCTAGACAGCAAGGTTATTACATGAGTTCTGATGTTTTTAAACAATTCACTAATCATGATGGAAAGTTCAAAGAAAATCACACTAATGATGTGCAAGGACTTCTTTCTCTTTATGAAGCTGCACATATGAGAGTTCATGATGAAGAAATTCTTGAAGAAGCTCTGATTTTTACTACTACACATCTTGAAAGTGTTATTCCAAACCTTAGTAATTCATTGAAAGTTCAAGTGACTGAAGCTTTGAGTCATCCAATTAGAAAAGCAATTCCAAGAGTTGGAGCTAGAAAGTATATTCATATCTATGAAAATATTGGTACTCATAATGATTTGTTATTGAAATTTGCTAAGCTTGATTTTAATATGTTGCAAAAACTTCATAGAAAGGAACTTAACGAACTTACATCTTGGTGGAAGGATCTTGATAGAGCTAACAAATTTCCTTATGCTAAAGATAGGTTGGTTGAAGCATATTTTTGGACTGTTGGTATCTATTTTGAACCACAGTATTCTCGTAGTAGGTCACTTGTTACTAAAGTTGTGAAGATGAATTCTATTATCGATGATACATATGATGCTTACGCAACTTTTGATGAATTAGTTCTTTTCACTGATGCTATTCAAAGGTGGGATGAAGGAGCTATGGATTTGCTTCCAACTTATTTGAGACCAATCTACCAAGGATTGCTTGATGTTTTTAATGAGATGGAAGAAGTTTTAGCTAAAGAGGGTAAAGCTGATCATATATATTATGCTAAGAAAGAGATGAAAAAAGTTGCTGAAGTTTACTTTAAGGAAGCTGAATGGCTTAATGCTAATTATATTCCAAAGTGTGAAGAATATATGAAGAACGGATTAGTTTCCTCAACTGGTCCTATGTACGGTATTATTTCCCTTGTTGTGATGGAAGAGATTATTACTAAAGAAGCATTTGAATGGCTTACTAATGAGCCACTTATCCTTAGGGCTGCTTCAACAATTTGTAGATTGATGGATGATATGGCTGATCATGAGGTTGAGCAACAAAGAGGTCATGTTGCATCATTTGTTGAATGTTATATGAAAGAATATGGAGTTTCTAAACAAGAAGCATATGTTGAAATGAGGAAAAAGATTACTAATGCTTGGAAAGATATTAATAAGGAACTTTTGAGACCAACAGCTGTTCCAATGTTTATTCTTGAAAGATCATTGAATTTTAGTAGATTGGCTGATACATTTCTTAAGGATGATGATGGTTATACTAATCCTAAGTCTAAGGTTAAAGATCTTATTGCATCTTTGTTTGTTGAATCTGTTGATATATGA  **Codon optimized sequence of *CYP450*:**  ATGCAATTCTTTAATTTCTTTTCATTGTTTCTTTTTGTTTCATTTTTGTTTCTTTTTAAAAAGTGGAAAAATTCTAATTCTCAAACTAAGAGATTGCCACCAGGACCATGGAAGCTTCCAATTCTTGGTAGTATGCTTCATATGCTTGGAGGATTGCCACATCATGTTCTTAGAGATCTTGCTAAGAAGTATGGACCTATTATGCATTTGCAGTTGGGTGAGGTTTCTTTGGTTGTTATTTCTTCTCCAGGAATGGCTAAAGAGGTTTTGAAAACTCATGATCTTGCTTTTGCTAATAGACCACTTCTTGTGGCTGCTAAGATTTTTAGCTATAATTGTATGGATATTGCTCTTTCACCATATGGTAATTATTGGAGACAAATGAGAAAAATTTGTTTGCTTGAACTTCTTTCTGCTAAAAATGTTAAGTCTTTTAATTCTATAAGACAGGATGAGGTTCATAGAATGATTAAGTTCTTTAGGTCATCTCCAGGTAAACCAGTTAACGTTACTAAAAGAATTTCTCTTTTTACAAATTCAATGACTTGTAGATCTGCTTTTGGTCAAGAATATAAGGAACAAGATGAATTTGTTCAACTTGTTAAAAAAGTTTCTAATTTGATTGAAGGATTTGATGTAGCTGATATTTTTCCATCATTGAAATTTTTGCATGTTTTGACTGGTATGAAGGCTAAAGTGATGAATACTCATAATGAGCTTGATGCTATTCTTGAAAATATTATTAATGAGCATAAGAAGACATCTAAATCAGATGGAGAATCTGGAGGTGAAGGTATTATAGGAGTTTTATTAAGATTGATGAAAGAAGGAGGACTTCAATTTCCAATTACTAATGATAATATTAAGGCTATTATTTCAGATATTTTTGGTGGTGGTACTGAGACTTCTTCTACAACTATTAATTGGGCTATGGTTGAAATGATGAAAAATCCTTCTGTTTTTAGTAAAGCTCAGGCTGAAGTTAGAGAAATTCTTAGAGGTAAAGAAACTTTTGGTGAAATTGATGTTGAAGAGTTTAAATATCTTAAAATGGTTATTAAAGAAACTTTTAGATTACATCCTCCTTTACCTCTTCTTCTTCCTAGAGAATGTAGAGAAGAAATTGATCTTAATGGTTATACAATTCCACTTAAGACAAAGGTTGTTGTTAATGCTTGGGCTATGGGTAGAGATCCTAAGTATTGGGATGATGTTGAATCTTTTAAGCCAGAAAGATTTGAACATAATTCTATGGATTATATAGGTAATAACTATGAATATCTTCCATTTGGTTCTGGTAGGAGGATTTGTCCAGGTATTTCTTTTGGACTTGCTAATGTTTATTTTCCTTTGGCTCAACTTCTTAATCATTTTGATTGGAAGCTTCCAACAGGAATTAATCCTAGAAATTGTGATTTGACAGAAGCTGCAGGTGCTGCTTGTGCTAGAAAGAATGATTTGCATTTGATAGCTACTGCATATCAACATTGTGAAGAATAA |
| --- |

**Table S2.** Primers for vector construction

| **Gene Name** | **Primer** | **Primer Sequence** | **Vector** |
| --- | --- | --- | --- |
| *CBTS1* | CBTS1-L | AAACCATGGTGTCTCAATCTATTTCTC | pGADT7-CBTS1 |
|  | CBTS1-R | AAACCATGGTGTCTCAATCTATTTCTC |  |
|  | CBTS1-IL | GCAAAGATGGATAAAGCGATGTCTCAATCTATTTCTCC | pGADT7-CBTS1-His |
|  | CBTS1-IR | CTACGATTCATCTGCAGTCAGTGGTGGTGGTGGTGGTGTATATCAACAGATTCAACAAAC |  |
| *CYP450* | CYP450-L | AAAGAATTCATGCAATTCTTTAATTTCTTT | pGBKT7-CYP450 |
|  | CYP450-R | AAAGGATCCTTATTCTTCACAATGTTGAT |  |

**Note:** Sequence for 6×His-tag is underlined.

**Table S3.** Gradient mobile phase for UPLC assay

| **Number** | **Time** (min) | **Flow rate** (mL/min) | **ddH_2_O** (%) | **Acetonitrile** (%) |
| --- | --- | --- | --- | --- |
| 1 | Initiation | 0.3 | 20 | 80 |
| 2 | 2 | 0.3 | 40 | 60 |
| 3 | 4 | 0.3 | 60 | 40 |
| 4 | 6 | 0.3 | 80 | 20 |
| 5 | 8 | 0.3 | 100 | 0 |
| 6 | 9 | 0.3 | 100 | 0 |
| 7 | 11 | 0.3 | 80 | 20 |
| 8 | 13 | 0.3 | 60 | 40 |
| 9 | 14 | 0.3 | 40 | 60 |
